# Breast cancer cell line toxicity of a flavonoid isolated from *Baccharis densiflora*

**DOI:** 10.1186/s12906-021-03349-4

**Published:** 2021-07-02

**Authors:** Wendy Soria Sotillo, Santiago Tarqui, Xiaoli Huang, Giovanna Almanza, Stina Oredsson

**Affiliations:** 1 Department of Biology, Lund University, Lund, Sweden; 2 Molecular Biology and Biotechnology Institute, University Major of San Andres, La Paz, Bolivia; 3 Chemical Research Institute, University Major of San Andres, La Paz, Bolivia

**Keywords:** Flavonoids, Sideritoflavone, Breast cancer, Cytotoxicity, Cell cycle, Cell movement, Cancer stem cells

## Abstract

**Background:**

Flavonoids are compounds of interest in the search for new anti-cancer therapies. We have previously isolated the methoxyflavones 5,4′-dihydroxy-6,7,8,3′-tetramethoxyflavone (8-methoxycirsilineol), 5,4′-dihydroxy-6,7,8-trimethoxyflavone (xanthomicrol), and 5,4,'3′-trihydroxy-6,7,8-trimethoxyflavone (sideritoflavone) from *Baccharis densiflora*. Herein, we investigate the toxicity of these methoxyflavones in human breast-derived cell line. Our main aim was to focus on the cancer stem cell (CSC) sub-population of JIMT-1 breast cancer cells.

**Methods:**

Initially, dose response experiments yielding inhibitory concentration 50 (IC_50_) values were performed using MCF-7, HCC1937, and JIMT-1 breast cancer, and the MCF-10A normal-like breast cell lines to get an understanding of toxic ranges. Due to a clear difference in the toxicity of the flavones, only sideritoflavone was selected for further studies using the JIMT-1 cell line. Effects on the CSC sub-population was investigated using flow cytometry-based methods. A wound healing assay and digital holographic microscopy were used to investigate effects on cell movement. A reporter assay was used to study effects on signal transduction pathways and Western blot for protein expression.

**Results:**

The dose response data showed that 8-methoxycirsilineol was non-toxic at concentrations below 100 μM, that the IC_50_ of xanthomicrol was between 50 and 100 μM, while sideritoflavone was highly toxic with a single digit μM IC_50_ in all cell lines. Treatment of the JIMT-1 cells with 2 μM sideritoflavone did not selectively effect the CSC sub-population. Instead, sideritoflavone treatment inhibited the proliferation of both the non-CSC and the CSC sub-populations to the same extent. The inhibition of cell proliferation resulted in an accumulation of cells in the G_2_ phase of the cell cycle and the treated cells showed an increased level of γ-H2A histone family member X indicating DNA double strand breaks. Analysis of the effect of sideritoflavone treatment on signal transduction pathways showed activation of the Wnt, Myc/Max, and transforming growth factor-β pathways. The level of p65/nuclear factor kappa-light-chain-enhancer of activated Β cells was increased in sideritoflavone-treated cells. Cell movement was decreased by sideritoflavone treatment.

**Conclusions:**

Altogether our data show that the methoxyflavone sideritoflavone has favourable anti-cancer effects that may be exploited for development to be used in combination with CSC specific compounds.

**Supplementary Information:**

The online version contains supplementary material available at 10.1186/s12906-021-03349-4.

## Background

Breast cancer is the most common cancer in women worldwide and in 2018 approximately 2 million new cases were reported [1]. Much effort is put into finding sources of new unique compounds that can be used on their own, in combinations, and for synthesis of compounds with higher bioactivity. A group of compounds of interest in the search for new anti-cancer therapy is flavonoids [2]. These plant-based secondary metabolites are categorized into flavonols, flavones, catechins, flavanones, anthocyanidins, and isoflavonoids. Flavonoids are powerful antioxidants with anti-inflammatory and immune system benefits and based on these data, the anti-cancer properties of the compounds have become a new field of interest. Recent studies also suggest that the consumption of different fruits and vegetables decreases the cancer risk level at least by 20% [2, 3], presumably due to their antioxidant properties. However, available evidence from cell culture experiments suggests that many biological effects of flavonoids are related to their ability to modulate cell signalling pathways [4]. Flavonoids have been associated with effects on gene expression, mutagenesis, carcinogenesis, and cell death. They can intercalate in the DNA duplex and react with free radicals in order to protect DNA from oxidative damage [5].

The *Baccharis* genus has been reported as a plant group with several therapeutic properties [6]. Among them, the anti-inflammatory activity is correlated with the level of flavonoids found in the plants. A survey of current available chemical data suggests that flavonoids such as methylated flavones, and to a lesser extent flavonols, mainly aglycones, are the main classes of phenolic compounds that can be found in *Baccharis spp.* [7, 8]. Here we investigated the anticancer properties of three flavones isolated from *Bacharis densiflora*, previously identified as *Baccharis pentlandii* [7]*,* a shrub used in Bolivia for the treatment of rheumatic diseases, sprains, broken bones, and dislocations [9].

Human cancer-derived cell lines are fundamental models used in laboratories to study the biology of cancer and to perform pre-clinical testing of new potential anti-cancer agents. The use of cancer cell lines is essential for the development of new anti-cancer drugs, understanding their mechanisms of action, the resistance/sensitivity patterns of chemotherapeutics already used in cancer treatment, and the development of more targeted anti-cancer drugs [10]. Human breast cancer cell lines maintain some of the phenotypic and genotypic heterogeneities seen in a tumour [11]. In breast tumours and also in a majority of other cancer types, a rare sub-population of cancer cells that has the ability to form new phenotypically and genotypically identical tumours at distant sites has been identified [12]. These cells have been coined tumour initiating cells or cancer stem cells (CSCs) [13]. In breast cancer, the CSCs are identified based on different markers and properties of the cells [14, 15]. Targeting of CSCs has been suggested to be important in cancer cure even though they may just be a fraction of the bulk of cancer cells in a tumour.

Herein, we investigated how three methoxyflavones isolated from *B. densiflora* affect bulk cancer cell properties such as cell proliferation and cell migration but also CSC specific properties. Only one of the flavones was active at a single digit μM inhibitory concentration 50 (IC_50_), while the other two had IC_50_ values above 50 μM despite quite similar chemical structures. The active compound, the methoxyflavone sideritoflavone, inhibited cell proliferation and cell migration but the mechanism of action does not involve CSC specific effects and thus it may e.g. be combined with CSC specific compounds in new cancer treatment strategies.

## Methods

### Plant material

Aerial parts of *B. densiflora* Wedd, previously identified as *Baccharis pentlandii* DC, were collected on February of 2017 in Cota Cota (3422 m.a.s.l.; 16° 32,270′ S 68° 4016′ W), located in the outskirts of La Paz, Bolivia. The plant was identified by Esther Valenzuela at the JBLP (*Jardín Botánico del Herbario Nacional de Bolivia*) where the voucher specimen is kept (code Beck St G. 2858).

### Extraction and isolation of compounds

Dried leaves of *B. densiflora* (200 g) were crushed and macerated in 96% ethanol, in a mass-solvent ratio of 1:15, at room temperature, for 15 min. The ethanol extract was filtered and the solvent was evaporated under reduced pressure in a rota-evaporator at a temperature of 40 °C, to obtain 20.8 g of dry extract. The ethanol extract (2 g) was separated by column chromatography on Sephadex LH-20 using 96% ethanol as the mobile phase to give 5 mg of a pure flavonoid **2** (xanthomicrol) and 50 mg of a mixture of the two flavonoids **1** and **3**. The mixture was subjected to preparative HPLC using a column of reverse phase C18–100* 10 mm and 5 μm with 100 μL loop. The mobile phase was a solvent mixture of 50% acetonitrile, 49.9% H_2_O, and 0.1% HCO_2_H. A solution of the flavonoid mixture at a concentration of 30 mg/ml, was separated with a flow of 2.8 ml/min for 11 min and analysed at a wavelength of 360 nm, obtaining 15 mg of sideritoflavone **3** and 10 mg of 8-methoxicirsineleol **1**. The compounds were identified by NMR data which were compared with bibliographic data [7, 16–18].

### Compounds and stock solutions

The compounds were dissolved in 100% dimethyl sulfoxide (DMSO) at a concentration of 100 mM and kept at 4 °C. Controls were treated with the same final DMSO concentration as the DMSO concentration in treated cultures maximally at 0.1% DMSO depending on the assay and chosen concentration.

### Cell lines and culture conditions

The JIMT-1 human breast carcinoma cell line (ACC589) was purchased from the German Collection of Microorganisms and Cell Cultures (Braunschweig, Germany). The normal-like breast epithelial MCF-10A cell line (CRL-10317), the cancer cell lines MCF-7 (HTB-22) and HCC1937 (CRL-2336) were purchased from American Type Culture Collection (Manassas, VA, USA). The cells were tested negative for mycoplasma (Eurofins, Konstanz, Germany).

The JIMT-1 cells were routinely cultured at 37 °C in a humidified incubator with 5% CO_2_ in air. The cells were cultured in DMEM/Ham’s F-12 medium supplemented with 10% fetal bovine serum (FBS), glutamine (2 mM), non-essential amino acids (1 mM), insulin (10 μg/ml), penicillin (100 U/ml), and streptomycin (100 μg/ml).

MCF-10A, MCF-7, and HCC1937 cell lines were cultured in RPMI 1640 medium (VWR) supplemented with 10% heat-inactivated FBS (VWR, Lund, Sweden), glutamine (2 mM), 1 mM non-essential amino acids (VWR), 10 μg/ml insulin (Sigma-Aldrich, Stockholm, Sweden), and 100 U/ml penicillin/100 μg/ml streptomycin (VWR). The MCF-10A cells were also supplemented with 20 ng/ml epidermal growth factor (Sigma-Aldrich), 50 ng/ml cholera toxin (Sigma-Aldrich), and 250 ng/ml hydrocortisol (Sigma-Aldrich). Finally, the HCC1937 medium was supplemented with 20 ng/ml epidermal growth factor (Sigma-Aldrich) besides the mentioned supplements.

### Dose response assay

For the dose response assay, cells were detached by trypsinization and counted in a hemocytometer. Cells were plated in 180 μl medium into 96-well flat-bottomed tissue culture plates followed by overnight incubation at 37 °C in the CO_2_ incubator. Cells were incubated in the presence of the compounds for 72 h at concentrations from 0.1 to 100 μM. After 72 h of compound exposure, 20 μl of 3-(4,5-dimethylthiazolyl-2)-2,5-diphenyltetrazolium bromide (MTT) (Sigma-Aldrich) solution (5 mg/ml in PBS) was added to each well, and the plates were returned to the CO_2_ incubator for 1 H*. *The medium with MTT was removed and the formazan crystals, formed by reduction in live attached cells, were dissolved in 100 μl DMSO. The plates were gently swirled at room temperature for 10 min. The absorbance was measured at 540 nm using a Multiskan™ FC microplate photometer and the software SkanIt for Multiskan FC 3.1. Ink, both from Thermo scientific (Waltham, Massachusetts USA). The results were analysed using GraphPad 6 computer software (La Jolla, California, USA). The IC_50_ values with 95% confidence interval are based on at least 3 dose-response experiments.

### Western blot analysis

Cells were seeded at a density of 300,000 cells in 5 ml medium in Petri dishes with 5 cm diameter. They were then incubated for 24 h to allow cell attachment. Then sideritoflavone was added at 2 or 2.5 μM concentrations. Cells were collected 72 h later by Accutase™ (Sigma, Sweden AB) treatment, resuspended, counted, and pelleted. The dry pellets were stored at − 80 °C until use. The pellets were then diluted in sample buffer (62.5 mM Tris-HCl (pH 6.8), 20% glycerol, 2% sodium dodecyl sulfate, 5% β-mercaptoethanol, and 1% NP-40; 100,000 cells/15 μl). The samples were sonicated twice for 20 s, boiled for 7 min, and stored at − 20 °C until further use. Pre-cast polyacrylamide Mini-PROTEAN® TGX™ Precast Gels (4–20% acrylamide Bis-Tris) were loaded with 15 μl of prepared sample per lane. Western blot and electrophoresis were performed in a Bio-Rad electrophoresis and blotting system (Bio-Rad, Hercules, California, USA). Electrophoresis was performed at 150 V for 5 min and at 300 V for 15 min in a Tris-glycine buffer. Then, the gels were blotted onto nitrocellulose membranes using a semi-dry Trans-Blot® Turbo™ Transfer System (Bio-Rad, Hercules, California, USA). The membranes were blocked in 5% bovine serum albumin (BSA) (Sigma-Aldrich, Copenhagen, Denmark) and 1% Tween 20 (Sigma-Aldrich) in PBS and incubated with primary antibodies against cyclin B1 (1:1000) (Santa Cruz Biotechnology, Texas, USA, sc-595), total p65 (1:1000) (Abcam, Cambridge, UK, ab76311), γ-H2A histone family member X (γ-H2AX) (1:1000) (Cell Signalling, Massachusetts, USA, # 2577), β-catenin (1:500) (BD Transduction laboratories™, CA, USA, 610154), or β-actin (1:500) (Abcam, Cambridge, UK). All antibodies were diluted in PBS containing 5% BSA and 0.1% Tween 20. After incubation with horseradish peroxidase (HRP)-conjugated swine anti-rabbit or HRP-conjugated goat anti-mouse secondary antibodies (Dako, Glostrup, Denmark) at room temperature, the membranes were exposed to enhanced chemiluminescent solution (GE Healthcare, Buckinghamshire, UK) to detect the protein bands. Data were collected and analysed using Quantity One software (Bio-Rad, Hercules, California, USA). The intensities of the bands were determined by densitometric scanning.

### Reporter assay for analysis of 10 signaling pathways

The Cignal Finder Cancer 10-Pathway Reporter Array (plate format) (Qiagen, Hilden, Germany) was used to simultaneously analyse the effect of treatment with sideritoflavone on 10 signalling pathways. JIMT-1 cells (2 × 10^4^ cells) were plated in 100 μL of Opti-MEM® containing 10% FBS per well in the provided 96 well white assay plate containing reporters. After 24 h, the cells were transfected overnight using Attractene. Cells were then treated for 24 h with 2 μM sideritoflavone. Firefly and Renilla signals were detected using Dual-Glo luciferase detection reagents (Promega, Wisc., USA) according to the manufacturer’s instructions. Renilla luciferase was used as the internal transfection control. Firefly luciferase levels were normalized to Renilla luciferase levels to generate a measurement of relative luciferase units. The results are presented as percentage luciferase activities normalized to transfected control JIMT-1 cells. The experiment was performed two times with three independent samples in each experiment.

### Scrape wound healing

The scrape wound healing assay that determines effects on directed cell migration was performed as described by Huang et al. [19]. Sideritoflavone was added to the final concentrations of 2 or 2.5 μM.

### Phase holographic imaging

Phase holographic imaging was used to monitor cell motility and cell proliferation. The JIMT-1 cells were seeded at a density of 135,000 cells per well in a 6-well plate. Cells were incubated for 24 h to let them attach to the bottom of the plate. Sideritoflavone was added to a concentration of 2 μM or 2.5 μM. The standard lid was replaced by HoloLid™ (71,110 PHI), and the 6-well plate placed on the motorized stage of a HoloMonitor® M4 (Phase Holographic Imaging AB (PHI), Lund, Sweden) placed in a CO_2_ incubator for cell culturing. Images were acquired with the software App Suite™ 2 (PHI) at three positions per well, every 5 min for 72 h. The experiment was repeated three times with two independent cultures for each treatment.

### Cell cycle phase distribution

Cells were seeded at a density of 300,000 cells in 5 ml medium in Petri dishes with 5 cm diameter. They were then incubated for 24 h to allow cell attachment. Then, sideritoflavone was added at 2 or 2.5 μM concentrations. Cells were collected 72 h later by Accutase treatment and resuspended in PBS containing 1% adult bovine serum. The cell concentration was determined by counting in a hemocytometer and a volume corresponding to 0.4 × 10 ^6^ cells was centrifuged at 600 *g* for 5 min at 4 °C to pellet the cells. The cells were fixed in 70% ethanol and stored at − 20 °C. The fixed cells were washed once with PBS, and after pelleting, the cells were resuspended in propidium iodide (PI)-nuclear isolation medium (PBS containing 100 μg/ml PI (Sigma-Aldrich), 0.6% NP-40 (Sigma-Aldrich), and 100 μg/ml ribonuclease A (Sigma-Aldrich) for at least 30 min at room temperature or overnight at 4 °C. The procedure removes the cell membrane and thus cells in mitosis were not included in the analysis. The method does not allow distinction between G_0_ and G_1_ phase cells as they have the same DNA content. The samples were analysed using a BD Accuri C6 flow cytometer (BD Biosciences, San Jose, CA, USA). The data were analysed using the MultiCycle software (Phoenix Flow Systems, San Diego, CA, USA).

### CD24/CD44 and ALDH analysis

The identification of the cell surface markers CD24 and CD44 and the analysis of ALDH positive cells was performed as described by Huang et al. [19]. Sideritoflavone was added to the final concentrations of 2 or 2.5 μM.

### Statistical analysis

A one-way ANOVA was used to determine if there was a significant difference between control and treatment means, and a Dunnet post-test was used to compare the difference between individual groups and control for all assays. GraphPad Prism version 6.00 for Windows, (GraphPad Software, La Jolla California USA) was used to run all statistical analyses. Treatment groups were considered significantly different if the *p* value was < 0.05.

## Results

### Dose response experiments

**Fig. 1:**
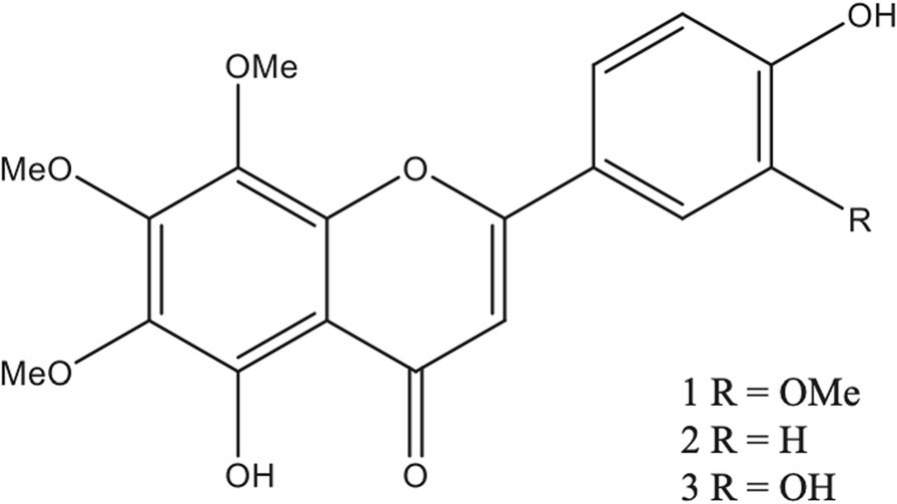
Polymethoxyflavones isolated from *Baccharis densiflora*. 1) 5,4′-dihydroxy-6,7,8,3′-tetramethoxyflavone (8-methoxycirsilineol). 2) 5,4′-dihydroxy-6,7,8-trimethoxyflavone (xanthomicrol). 3) 5,4,'3′-trihydroxy-6,7,8-trimethoxyflavone (sideritoflavone)

**Fig. 2:**
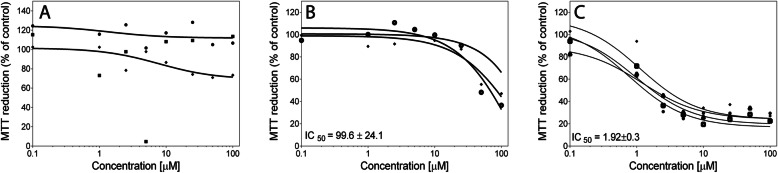
Dose response curves obtained after treating JIMT-1 breast cancer cells with **A** 8-methoxycirsilineol, **B** xanthomicrol, and **C** sideritoflavone. The cells were seeded in the wells of 96-well plates and 24 h later the compounds were added at concentrations shown in the figures. The dose response was evaluated with an MTT assay after 72 h of incubation with compound. Each curve shows an independent experiment with mean for *n* = 6 in each data point. The IC_50_ values were deduced from the MTT-based dose-response curves. The IC_50_ data are presented as the mean ± SD from 2 to 4 experiments

Dose response experiments were performed treating the cell lines with the three flavones 8-methoxycirsilineol, xanthomicrol, or sideritoflavone containing differences in the phenyl ring shown in Fig. 1. Among them, only sideritoflavone which has *ortho* dihydroxy groups (type catechol) in C-3′ and C-4′ showed cytotoxicity at low single digit μM concentrations with an IC_50_ of 1.9 ± 0.3 μM for JIMT-1 cells (Fig. 2). Treatment with 8-methoxycirsilineol, which has an OMe in C-3′ and an *ortho* dihydroxy group in C-4′, up to a concentration of 100 μM did not give a cytotoxic effect and treatment with xanthomicrol that only has one OH in C-4′ showed lower cytotoxicity with an IC_50_ of 99.6 ± 24.1 μM. Sideritoflavone treatment was also toxic to MCF-7 (IC_50_ 4.9 ± 1.7 μM) and HCC1937 (IC_50_ 4.6 μM) breast cancer cells and MCF-10A (IC_50_ 6.7 ± 0.9 μM) normal breast epithelial cells while methoxycirsilineol and xanthomicrol were much less toxic (IC_50_ ≥ 100 μM) (supplemental information Fig. S1).

### Effects on cell proliferation and the cell cycle phase distribution

Since we have used the JIMT-1 cells for intensive investigation of the effect of treating with different compounds on the CSC sub-population, cell movement, and molecular aspects, we decided to use only this cell line for in depth studies [19–24]. In addition, based on the dose response data, we decided to only proceed with sideritoflavone as effects at low doses more easily achievable in the body is important in cancer treatment. We chose to use the doses 2 and 2.5 μM, the lower dose being close to IC_50_ and a slightly higher dose to get an understanding of the sensitivity to the compound at small differences in treatment concentration.

**Fig. 3:**
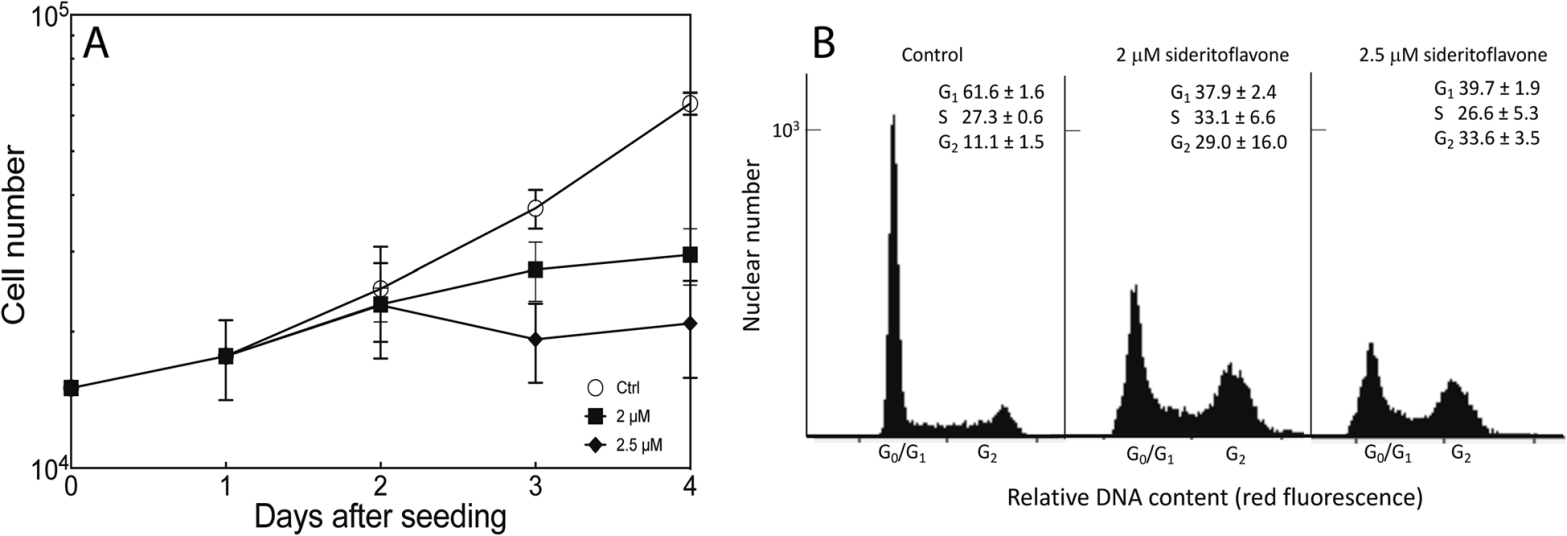
Sideritoflavone inhibits cell proliferation and affects cell cycle phase distribution of JIMT-1 breast cancer cells. Cells were seeded day 0, and the compound was added 1 day after seeding to reach the final the concentrations of 2 μM or 2.5 μM. Control was treated with 0.1% DMSO which was the same DMSO concentration as in treated cultures. **A** The cell number was determined using a Holomonitor® M4. Data are presented as the mean of 6 independent samples from 3 independent experiments and bars ± SD. **B** At 72 h of treatment, cells were sampled for analysis of cell cycle phase distribution using flow cytometry of cells labelled with propidium iodide. The numbers show the cell cycle phase distribution in % for *n* = 3 ± SD

Figure 3A shows the effect on cell proliferation of treating JIMT-1 cells with 2 or 2.5 μM sideritoflavone in comparison to control. A slight inhibition of cell proliferation was observed 1 day after addition of the compound and between 2 and 3 days of treatment, the cell number did not change in the treated cultures while control continued to proliferate. At 2 days of treatment, the cell number in cultures treated with 2 and 2.5 μM sideritoflavone were 73 and 51% of control and at 3 days of treatment, 46 and 33% of control, respectively. Thus, the 2 μM concentration at 72 h of treatment resulted in an effect on cell number in percent, similar to the IC_50_ obtained from the dose response curve. The MTT data are assumed to reflect cell number which is not always the case depending on effects of a compound on the reduction of MTT in mitochondria.

Sideritoflavone treatment resulted in decreased number of cells in the G_0_/G_1_ phase while there was an increase in the G_2_ phase (Fig. 3B).

### Cell movement

**Fig. 4:**
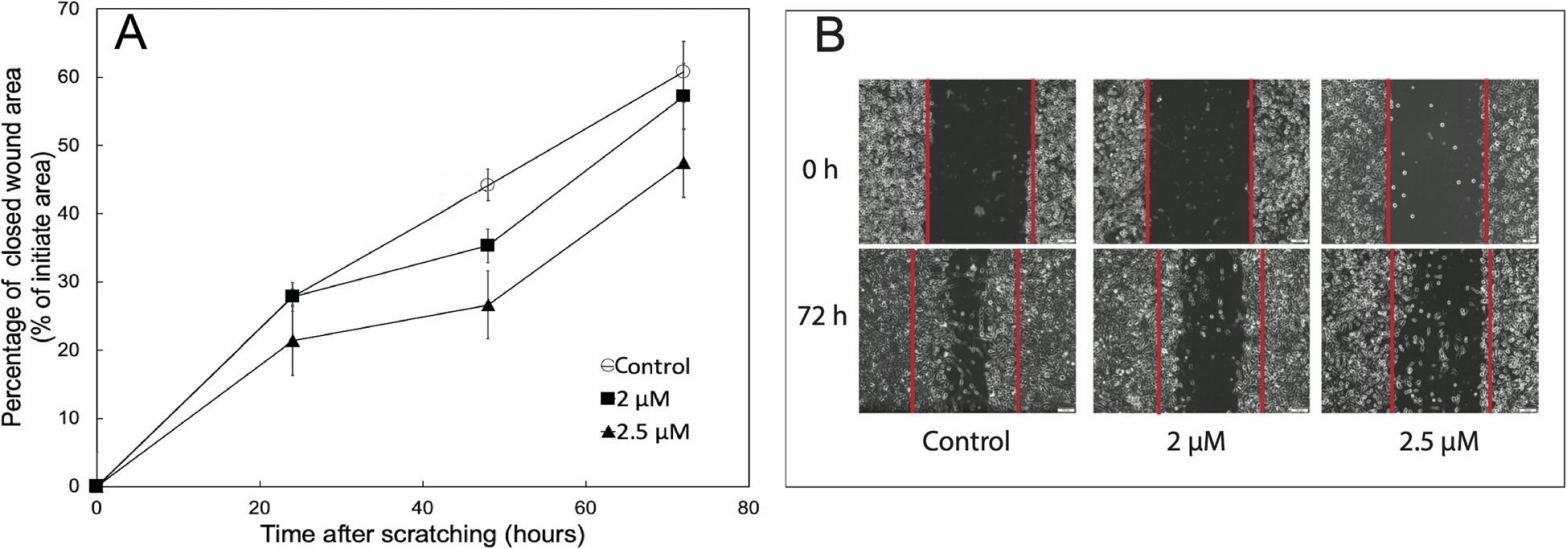
Treatment with sideritoflavone reduces cell migration in a wound healing assay. Twenty-four hours after seeding of cells at high density, a wound scratch was made in the cell layer, and after washing, the cells received complete medium without FBS and with the treatment at indicated concentrations. The wound scratch was photographed at 0 h i.e. directly after the scratching and addition of compound, and at 24, 48, and 72 h thereafter. **A** Quantified wound healing. Data points are the mean of three independent cultures ± SEM. **B** Representative images. The red lines delineate the initial wound area

As the ability to migrate is important for cancer cell metastasis, we investigated the effect of sideritoflavone treatment on the migration of the JIMT-1 cells in a wound healing assay using serum free medium to minimise the influence of cell proliferation on the data (Fig. 4). In this assay, the cells move from an edge into a wound area. Sideritoflavone treatment reduced the directed migration of JIMT-1 cells into the wounded area (Fig. 4).

**Fig. 5:**
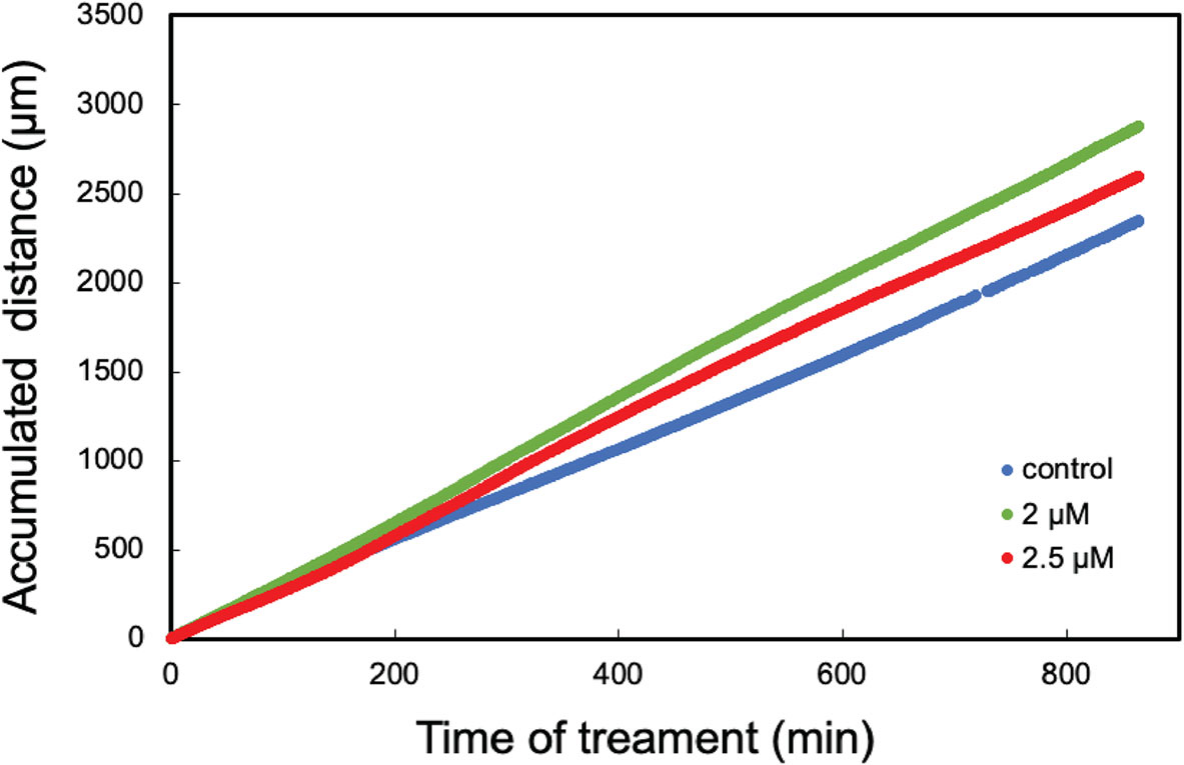
Treatment of JIMT-1 cells with sideritoflavone increases motility determined from time-lapse movies obtained using a digital holographic microscope. Cells were seeded and 1 day later (time 0 in the figure), sideritoflavone was added to the final concentrations of 2 or 2.5 μM. Images were taken every 5 min. Motility was evaluated using the software App Suite™ 2 (PHI). The data are from three independent experiments with *n* = 2 in each. The data points are very close, thus forming a line

To obtain further details, cell motility was studied in real time using a phase holographic microscope where images were taken every 5 min. The cells were cultured in their regular medium containing FBS in the absence or presence of 2 or 2.5 μM sideritoflavone. Cell motility was evaluated using App Suite™ (Fig. 5). Treatment with sideritoflavone increased the motility of the cells. Motility is defined as the total accumulated distance a cell has moved over the time of tracking. Motility is not a measure of how far the cells are migrating from a starting point but is a measure of how the cells are moving around. Thus, a cell can have low migratory ability but may be moving around in a small spot resulting in high motility. This is the case with JIMT-1 cells treated with sideritoflavone. The time laps movies are found in supplementary information (Time-lapse movie S1, S2, and S3 for control, and 2 and 2.5 μM sideritoflavone, respectively).

### Treatment with sideritoflavone does not affect the cancer stem cell sub-population

**Fig. 6:**
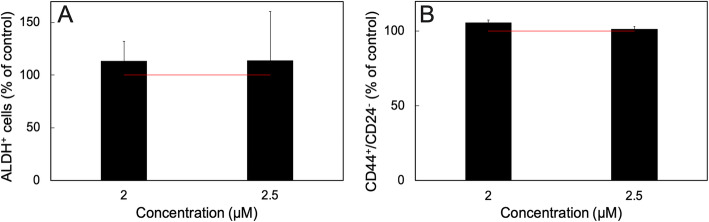
Treatment of JIMT-1 breast cancer cells with sideritoflavone for 72 h does not reduce the CSC sub-population. **A** The CD44^+^/CD24^−^ population and **B** the ALDH^+^ populations were evaluated by flow cytometry. Data are presented as the mean ± SE for *n* = 3

The JIMT-1 breast cancer cell line contains a sub-population of cells with a CSC phenotype defined as CD44^+^/CD24^−^ and aldehyde dehydrogenase positive (ALDH^+^) [19–24]. Treatment of JIMT-1 cells with e.g. salinomycin [19, 22, 24] or the sesquiterpene lactones damsin and ambrosin [20] decreases this sub-population as evaluated by determining the effect of treatment on the CD44^+^/CD24^−^ and aldehyde ALDH^+^ populations by flow cytometry. When we investigated the effect of sideritoflavone treatment on the CD44^+^/CD24^−^ and ALDH^+^ cells, there was no specific reduction of these sub-populations (Fig. 6). CSCs form colonies when seeded at cloning density in serum free medium [25]. In one experiment investigating the effect of sideritoflavone treatment on the colony forming efficiency of JIMT-1 cells, we found no effect on this population (not shown). Thus, we did not repeat the colony forming experiment as the CD44^+^/CD24^−^ and ALDH^+^ analyses already showed no effect on the CSCs.

### Signalling pathways affected by sideritoflavone treatment

**Fig. 7:**
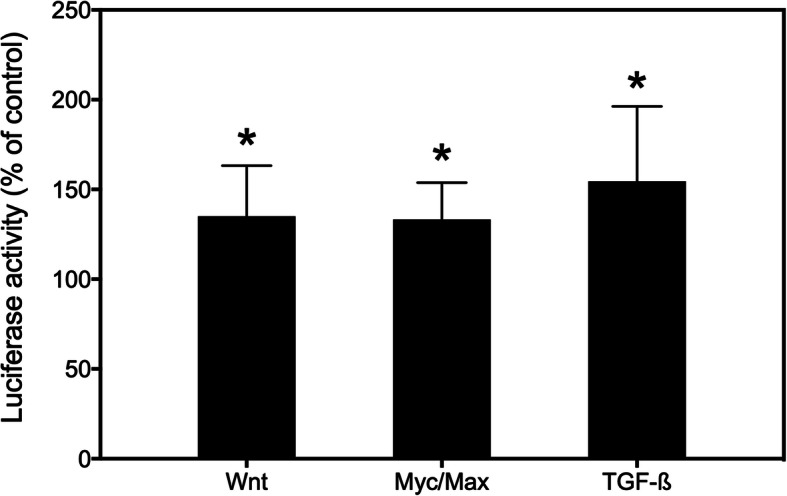
Effect of treating JIMT-1 breast cancer cells with sideritoflavone on signal transduction pathways. The Cignal Finder Reporter Array (Qiagen) was used to investigate the effect after 24 h of treatment with 2 μM sideritoflavone. The data show the mean of two experiments compared to control with three independent samples in each ± SEM

We then applied a Cignal Finder Reporter Array (Qiagen) to screen for effects on signal transduction pathways in JIMT-1 cells treated with 2 μM sideritoflavone for 24 h. The data show that treatment with sideritoflavone significantly increased Wnt, Myc/Max, and transforming growth factor-β (TGF-β) signalling (Fig. 7).

### Specific proteins affected by sideritoflavone treatment

**Fig. 8:**
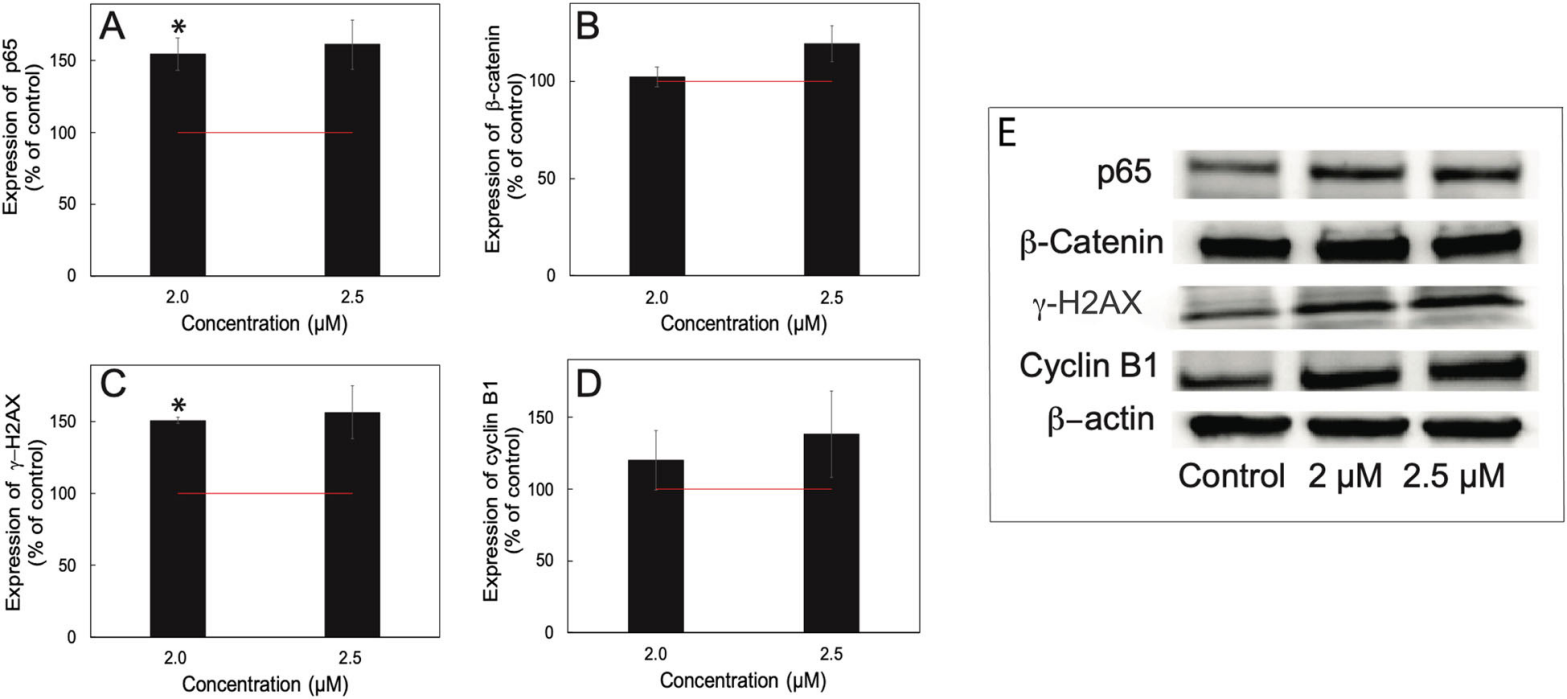
The effect of treatment with sideritoflavone for 72 h on protein levels of **A** p65/NF-κΒ, **B** β-catenin, **C** γ-H2AX, and **D** cyclin B1. The expression of the proteins was analysed by Western blot after 72 h of treatment at the concentrations shown in the figures. The data were analysed by densitometric scanning and expressed in % of control. *n* = 3–4 independent experiments ± SEM. The figure includes representative Western blots. The uncropped Western blots are found in Supplementary information Figure S2

Treatment with flavonoids has been reported to increase the p65/NF-κΒ level [26] as well as decrease p65/NF-κΒ activity [27]. Here we found that treatment with sideritoflavone increased the cellular level of p65 investigated by Western blot (Fig. 8A).

The increase in Wnt signaling observed in the pathway analysis lead us to investigate the level of β-catenin, however, there was no significant effect on the level as determined by Western blot (Fig. 8B).

Since the DNA histograms indicate DNA damage (broadening of peaks), we decided to investigate γ-H2AX, a marker for DNA double strand breaks [28]. The level of γ-H2AX was increased in cells treated with sideritoflavone compared to control (Fig. 8C). Also, the accumulation of cells in the G_2_ phase of sideritoflavone-treated cells explains the increased cyclin B1 level (Fig. 8D). Cyclin B1 levels rise in S phase and peak at the G_2_/M boundary [29].

## Discussion

In the present work, we have investigated the toxicity of three methoxy flavones isolated from *B. densiflora* [7]. The flavones 8-methoxycirsilineol, xanthomicrol, and sideritoflavone have similar structures but very different toxicity profiles in our study. 8-Methoxycirsilineol did not show toxicity up to 100 μM concentration while sideritoflavone was toxic in the single digit μM range. Here we find that sideritoflavone is 50 times more toxic to JIMT-1 cells compared to xanthomicrol despite the fact that the compounds only differ by one hydroxyl group. In sideritoflavone, the phenyl ring contains one catechol fragment and in xanthomicrol the phenyl ring possess just one OH in C-4′. Grigalius and Petrikaite [30] evaluated seventeen flavones on three different human cancer cell lines, A549 lung cancer cells, the MCF-7 breast cancer cells, and U87 glioblastoma cells. The most active compounds were those with catechols in the phenyl ring which was considered the most important structural feature of flavonoids for antioxidant and anti-cancer activities [31, 32]. To the best of our knowledge there are no studies comparing the toxicity of the three compounds tested here and overall, there are few studies related to toxicity of these compounds specifically.

Moghaddam et al. [32], compared the cytotoxicity of xanthomicrol with 8 other flavonoids in 6 different cell lines using an MTT assay. For one of the cell lines (human gastric adenocarcinoma) the IC_50_ for xanthomicrol was 13 μM while the IC_50_ was in the range of 94–161 μM in the other cell lines. Here we found an IC_50_ of almost 100 μM for the JIMT-1 cell line and the IC_50_ was even higher for the MCF-7 and HCC1937 cell lines. Sideritoflavone was considerably more toxic than xanthomicrol in both the cancer cells (JIMT-1, MCF-7, and HCC1937 with IC_50_ of 1.9, 4.9, and 4.6 μM, respectively) and in the normal-like MCF-10A cells (6.7 μM). Comparing IC_50_ values of treating JIMT-1 cells and MCF-10A cells with sideritoflavone shows that the former cell line is about 3.5 times more sensitive. To the best of our knowledge, we have only found two studies presenting IC_50_ for sideritoflavone [33, 34]. Novelo et al. [33], report an IC_50_ of 3 μM in human oralepidermoid carcinoma cells and Beutler et al. [34], an IC_50_ of 4 μM for the human 60 cell line cytotoxicity screen. Because of these data, we decided to only proceed with further studies treating JIMT-1 breast cancer cells with sideritoflavone.

The IC_50_ for sideritoflavone obtained using the MTT assay, which is only an indirect determination of cell number was confirmed by an investigation of the effect of sideritoflavone on the cell number through a growth curve experiment. Effects on cell proliferation may result in changes in the cell cycle phase distribution and that is also found for JIMT-1 cells treated with sideritoflavone. The growth inhibitory effect of sideritoflavone was accompanied by an increase in the G_2_ phase population and a decrease in the G_1_ phase population while the S phase population was almost unchanged. A similar change in cell cycle phase distribution has been found for other cells treated with different flavones [35].

The flavone quercetin has been shown to bind to DNA by intercalation [36]. Intercalation in DNA has been shown to cause DNA double strand breaks [37] and an increased level of γ-H2AX is a biomarker for DNA double strand breaks [28]. Here we found that the level of γ-H2AX increased in sideritoflavone-treated cells compared to control investigated by Western blot. Further studies are needed to confirm DNA double stand breaks, however, that is not within the scope of this research.

The accumulation of cells in G_2_ phase caused by sideritoflavone treatment may be a response to induction of DNA double strand breaks. DNA double strand breaks are known to induce a multitude of pathways [38, 39], among which is the c-Myc/Max pathway. The c-Myc/Max pathway was significantly activated in sideritoflavone-treated cells compared to control.

The movement of cells can be described by different terms. Cell motility is regarded as random cell movement, while cell migration is a directed movement due to different signals. Cancer cell migration and invasion may result in the formation of distant metastasis while an increase in cell motility is just a local phenomenon. Here we found that sideritoflavone treatment increased the local motility of JIMT-1 cells, however, directed cell migration evaluated in a wound healing assay was inhibited. Thus, an increased motility does not per se result in increased migration in the wound healing assay. The same has been found when treating JIMT-1 cells with the CSC inhibiting compound salinomycin [40]. Increased cell migration and motility without a clear distinction of the terms have been associated with activation of the TGF-β signaling pathway and with increased p65/NF-κΒ [41]. Here we did find an activation of the TGF-β signaling pathway and an increased level of p65/NF-κΒ in sideritoflavone-treated cells with increased motility and decreased migration. Obviously, more studies are needed to understand the connection between motility and migration and the molecular mechanisms involved.

CSCs have been suggested to be the main cause of metastasis and cancer death due to their resistance to treatment and to their ability to migrate [42]. Thus, there is an intensive search for CSC targeting compounds [43, 44]. Treatment with sideritoflavone did not specifically target the CSC population but resulted in a decrease in both the CSC and non-CSC populations to the same degree, as the proportion did not change when the cell number decreased. It may be favourable to combine a specific CSC targeting compound like salinomycin [15] or sesquiterpene lactones [16] with sideritoflavone.

## Conclusions

In conclusion, we show here considerable differences in toxicity among the methoxyflavones 8-methoxycirsilineol, xanthomicrol, and sideritoflavone isolated from *B. densiflora* despite the fact that they have similar chemical structures. Sideritoflavone was markedly more toxic than xanthomicrol while 8-methoxycirsilineol was not toxic up to a 100 μM concentration, showing the importance of a catechol fragment in the phenyl ring of these methoxyflavones. We have not elucidated the exact mechanism by which sideritoflavone inhibits cell proliferation but our data together with reports by others, suggests that DNA intercalation may be the cause for the toxic response. Sideritoflavone treatment inhibited directed cell migration which is a favourable trait for a cancer treatment drug. Sideritoflavone inhibited the non-CSCs and the CSCs to the same extent. Thus, it may be favourable to combine sideritoflavone with CSC targeting compounds.

## Supplementary Information

**Additional file 1: Figure S1.** Dose response curves obtained after treating MCF-10A normal-like breast epithelial cells and MCF-7 and HCC1937 breast cancer cells with 8-methoxycirsilineol (only MCF-10 and MCF-7), xanthomicrol, or sideritoflavone. The cells were seeded in 96-well plates and 24 hours later the compounds were added at concentrations shown in the figures. The dose response was evaluated with an MTT assay after 72 hours of incubation with compound. Each curve shows an independent experiment with mean for *n* = 6 in each data point. The IC_50_ values were deduced from the MTT-based dose-response curves. The IC_50_ data are presented as the mean ± SD from 1–3 experiments with *n* = 6 in each.

**Additional file 2: Figure S2.** Representative un-cropped Western blots used for the cropped Western blot bands shown in Fig. 8.

**Additional file 3.**

**Additional file 4.**

**Additional file 5.**

## Data Availability

All original data are available upon request.

## Abbreviations

ALDH: Aldehyde dehydrogenase
BSA: Bovine serum albumin
CSCs: Cancer stem cells
γ-H2AX: γ-H2A histone family member X
IC_50_: Inhibitory concentration 50
MTT: 3-(4,5-dimethylthiazolyl-2)-2,5-diphenyltetrazolium bromide
NF-κΒ: Nuclear factor kappa-light-chain-enhancer of activated Β cells
PHI: Phase holographic imaging
TGF-β: Transforming growth factor-β
