# Supplementary material for: Breast cancer cell line toxicity of a flavonoid isolated from Baccharis densiflora
Source: BMC Complement Med Ther. 2021 Jul 2;21:188. doi: 10.1186/s12906-021-03349-4 (PMC8254278; doi:10.1186/s12906-021-03349-4)
Supplement: Supplementary file 1 — Additional file 1: Figure S1. Dose response curves obtained after treating MCF-10A normal-like breast epithelial cells and MCF-7 and HCC1937 breast cancer cells with 8-methoxycirsilineol (only MCF-10 and MCF-7), xanthomicrol, or sideritoflavone. The cells were seeded in 96-well plates and 24 hours later the compounds were added at concentrations shown in the figures. The dose response was evaluated with an MTT assay after 72 hours of incubation with compound. Each curve shows an independent experiment with mean for n = 6 in each data point. The IC50 values were deduced from the MTT-based dose-response curves. The IC50 data are presented as the mean ± SD from 1–3 experiments with n = 6 in each. [file 12906_2021_3349_MOESM1_ESM.pdf]

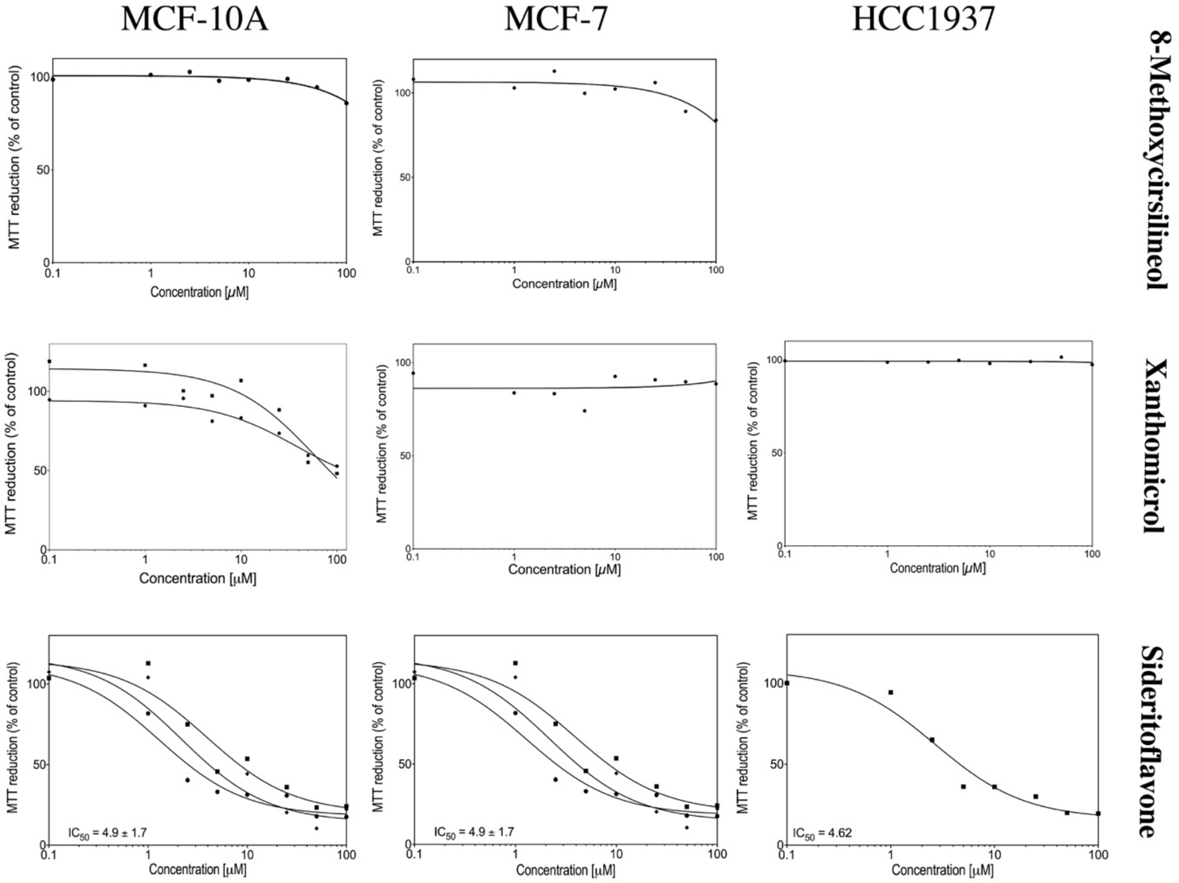

Supplementary Figure S1. Dose response curves obtained after treating MCF-10A normal-like breast epithelial cells and MCF-7 and HCC1937 breast cancer cells with 8-methoxycirsilineol (only MCF-10 and MCF-7), xanthomicrol, or sideritoflavone. The cells were seeded in 96-well plates and 24 hours later the compounds were added at concentrations shown in the figures. The dose response was evaluated with an MTT assay after 72 hours of incubation with compound. Each curve shows an independent experiment with mean for n = 6 in each data point. The IC_{50} values were deduced from the MTT-based dose-response curves. The IC_{50} data are presented as the mean ± SD from 1–3 experiments with n = 6 in each.
