# Supplementary material for: Breast cancer cell line toxicity of a flavonoid isolated from Baccharis densiflora
Source: BMC Complement Med Ther. 2021 Jul 2;21:188. doi: 10.1186/s12906-021-03349-4 (PMC8254278; doi:10.1186/s12906-021-03349-4)
Supplement: Supplementary file 2 — Additional file 2: Figure S2. Representative un-cropped Western blots used for the cropped Western blot bands shown in Fig. 8. [file 12906_2021_3349_MOESM2_ESM.docx]

Figure S2. Representative un-cropped Western blots used for the cropped Western blot bands shown in Figure 8.
